# The Influence of Chitosan on the Oral Bioavailability of Acyclovir—a Comparative Bioavailability Study in Humans

**DOI:** 10.1007/s11095-014-1613-y

**Published:** 2015-01-22

**Authors:** Marlies Kubbinga, Mai Anh Nguyen, Petra Staubach, Steven Teerenstra, Peter Langguth

**Affiliations:** 1 Centre for Health Protection, National Institute for Public Health and the Environment, Bilthoven, The Netherlands; 2 Institute of Pharmacy and Biochemistry, Johannes Gutenberg University Mainz, Staudingerweg 5, 55128 Mainz, Germany; 3 Medicines Evaluation Board, Utrecht, The Netherlands; 4 Department of Dermatology, Clinical Research Center, University Medical Center, Mainz, Germany

**Keywords:** bioavailability, biopharmaceutics classification system, biowaiver, excipient interactions, pharmacokinetics

## Abstract

**Purpose:**

The effects of chitosan hydrochloride on the oral absorption of acyclovir in humans were studied to confirm the absorption enhancing effects reported for *in vitro* and rat studies, respectively.

**Methods:**

A controlled, open-label, randomized, 3-phase study was conducted in 12 healthy human volunteers. Zovirax 200 mg dispersible tablets co-administered with doses of 400 and 1000 mg chitosan HCl were compared with Zovirax only.

**Results:**

The expected increased absorption of acyclovir was not observed. On the contrary, mean area under the plasma concentration-time curve (AUC0-12 h) and maximal plasma concentration (C_max_) decreased following concomitant chitosan intake (1402 *versus* 1017 and 982.0 ng∙h/ml and 373 *versus* 208 and 235 ng/ml, respectively). In addition, T_max_ increased significantly in presence of 1000 mg of chitosan from 1 to 2 h.

**Conclusions:**

The results of this study in human volunteers did not confirm an absorption enhancing effect of chitosan. Reference values were comparable to literature data, whereas addition of chitosan resulted in significant opposite effects on C_max_, T_max_ and AUC. Additional studies are needed to investigate the cause of the discrepancy. The observed variability and complex potential interactions may complicate the use of chitosan HCl in oral pharmaceutical formulations.

**Electronic supplementary material:**

The online version of this article (doi:10.1007/s11095-014-1613-y) contains supplementary material, which is available to authorized users.

## INTRODUCTION

Permeation enhancement has been a research topic in pharmaceutics for decades. Increasing the absorption of drugs with low permeability may for instance aid the reduction of the variability in bioavailability as well as reduce the administered dose of an active substance. Examples of absorption modulators are acetylcystein, supposedly acting *via* reduction of the mucous layer by disrupting disulfide bridges; surfactants, polymers and chelating agents potentially interfering with the tight junctions, and substances interfering with absorption or efflux transporters (1–3).

Chitosan and its derivatives have been described as potential permeability enhancers acting *via* a disruptive effect on the tight junctions between the epithelial cells (4–9). Chitosan is a heteropolysaccharide derived from natural source; characterized by variable molecular weights, degrees of deacetylation (DD) and salt forms (10).

Acyclovir is a BCS class III substance that is predominantly absorbed *via* the paracellullar route and has a low and variable bioavailability of 10–30% (11,12). Some studies suggest the existence of a saturable carrier system or a limited absorption window. Most of the drug is renally excreted in untransformed state (13,14). Chitosan was shown to increase the *in vitro* permeability of acyclovir across Caco-2 monolayers and the absorption of acyclovir in the rat (15–17). In the absence of clinical studies in humans, the translation of these effects in cell culture and animal models to humans currently remains unclear. The aim of the study presented here was to test the effects of chitosan hydrochloride on the oral absorption of acyclovir in human volunteers.

## MATERIALS AND METHODS

### Materials

Zovirax 200 mg dispersible tablets (GlaxoSmithKline, Austrian license number 1-18043) were used as reference product. Chitosan hydrochloride (chitosan HCl) of pharmacopoeial quality was obtained from Heppe Medical, Halle; degree of deacetylation (DD) 93.05%, viscosity 1% in water at 20°C 5.9 mPas, molecular weight (MW) 30–400 kDa. It was dispensed into quantities of 400 and 1000 mg and labeled by Löwen Apotheke in Hochspeyer, Germany. The powders released by the Department of Pharmaceutical Technology and Biopharmaceutics of the Johannes Gutenberg University in Mainz, Germany. Acyclovir reference material was purchased from Fagron. All other materials were purchased from Sigma Aldrich.

### Study Design

A controlled, open-label, randomized, 3-phase study was conducted at the Clinical Research Center of the University Hospital in Mainz, Germany. The clinical trial protocol (EudraCT-Nr. 2010-023882-22) was approved by both the relevant Ethics Committee (Landesärztekammer Rheinland-Pfalz) and the German competent authorities (Bundesinstitut für Arzneimittel und Medizinprodukte, BfArM) and the study was performed in accordance with the Declaration of Helsinki. Adult, male and female healthy volunteers were included after a health check based on interview, blood pressure, blood parameters and ECG. Taking into account the variation coefficients as presented previously by Vergin *et al*. in a bioequivalence study with 200 mg acyclovir products and considering an estimated minimum effect size of 30% as potentially clinically relevant, a number of 12 volunteers was selected for this exploratory study (18). Participants did not take part in another clinical study in parallel and did not take part in one within the preceding 90 days. Other exclusion criteria were: alcohol abuse or medication dependency in anamnesis, known hypersensitivity to study medication, active liver disease or unexplained increased levels of serum transaminases, intake of prescription drugs in the last 1–2 months, a recent (90 days) history of cytomegalovirus or systemic herpes infection(s) or recurrent systemic infections of herpes viruses, pregnancy and lactation, renal dysfunction. The subjects received Zovirax on the first trial day and were then randomly assigned to either the sequence 1000 mg followed by 400 mg chitosan HCl or *vice versa*. This resulted in a 2-treatment (400 *vs* 1000 mg chitosan HCl), 2-sequence crossover design with pretreatment by Zovirax. This study design allowed evaluation of the pharmacokinetics of acyclovir, without potential interference of chitosan. A wash-out phase of 7 days was applied for each treatment.

Zovirax tablets were used as reference and the same tablets, co-administered with a known quantity of chitosan HCl, were used as test ‘formulations’. All products were dispersed in 100 mL water and administered with another 150 mL, resulting in a total volume of 250 mL water. Start concentrations of 1.6 g/L (0.16%) and 4.0 g/L (0.4%) chitosan HCl were thus obtained, in line with those described in literature (0.1%–0.5%) (15,16). The maximum chitosan dose was limited to 1 g in view of the practical feasibility of oral intake of the quantity as a single dose and the related limited relevance of higher quantities as a potential excipient in an actual solid oral dosage form.

Subjects entered the studies fasting for at least 9.5 h. The first meal was offered 4 h post-dose. The medication was administered with a total of 250 mL water; subjects had access to more water from 1 h post-dose on and consumed at least 1.4 L mineral water during the 12 h trial. Blood samples were collected prior to administration of the product(s) and at 0.25, 0.5, 0.75, 1, 1.5, 2, 2.5, 3, 4, 6, 8 and 12 h after dosing. Blood samples were centrifuged for separation of plasma and stored at or below −20°C prior to analysis.

### HPLC Assay

Plasma samples of 500 μl were prepared for analysis by solid phase extraction, using Oasis HLB 1 cc cartridges (30 mg) supplied by Waters. The column was washed with 500 ml water and then eluted with 500 μl acetonitrile. The extract was centrifuged at 14000 rpm for 10 min at −5°C and the concentration of acyclovir was determined by LC-MS/MS using a sample injection volume of 10 μl. The bioanalytical method was modified from previously reported methods (16,19,20). Validation data are included as supplementary material. The HPLC consisted of a Prontosil C18; 100*2,00 mm; 5 μm column, using an Agilent 1100 LC binary pump. A gradient elution was used with mobile phase A and B where A consisted of 15 mM ammonium acetate + 0.1375% formic acid at pH 3.5 and B was acetonitrile + 0.1375% formic acid. Details of the gradient:

**Table:** 

Step	Total Time(min)	Flow Rate(μl/min)	A (%)	B (%)
0	0.00	300	97.0	3.0
1	0.50	300	97.0	3.0
2	0.60	300	5.0	95.0
3	1.20	300	5.0	95.0
4	2.00	300	97.0	3.0
5	5.50	300	97.0	3.0

Detection took place by a triple quadrupole LC-MS/MS mass spectrometer, API 3000 manufactured by AB Sciex Instruments, using multiple reactions monitoring with transitions Q1/Q3: 255,992 → 151,873. Source temperature was 500°C, overall run time was 5.5 min. The method was linear in a range of 10 to 800 ng/ml, with a detection limit of 1 ng/ml. QC samples were analyzed with the plasma samples to monitor method accuracy and precision.

### Pharmacokinetic and Statistical Analysis

The data were analyzed using Microsoft Excel 2010 and IBM SPSS Statistics version 19. Maximum plasma concentration, C_max,_ and the time point at which this was measured, T_max,_ were manually selected from the data. The areas under the curve, AUC_(0-t)_ and AUC_(0-∞)_, were estimated using the linear trapezoidal method as available in PKSolver2.0 with automatic calculation of the terminal elimination slope using the regression with the largest adjusted R^2^ based on at least three of the last time points (21).

The complete dataset was analyzed for sequence or period effects on C_max_ and AUC using a general linear model with period, treatment, sequence and subject within sequence as fixed effects. The difference between related parameters was considered statistically significant if *p* ≤ 0.05.

Parametric 90% confidence intervals were calculated based on the univariate ANOVA of the mean ratio of test *versus* reference using log-transformed C_max_ and AUC data. An increase of the bioavailability of more than 30% was predefined as clinically significant. In addition, the products were evaluated based on the European Guideline on bioequivalence. Test and reference products were considered bioequivalent if the ln-transformed ratios of C_max_ and AUC and their confidence intervals were within the equivalence range of 80–125% (22).

T_max_ was analysed using the nonparametric Wilcoxon signed rank test, where *p* ≤ 0.05 was considered statistically significant.

### Tolerability

Tolerability was assessed by monitoring electrocardiogram and blood parameters before and after the study and by subject interviews during the study.

## RESULTS

### Tolerability

The formulations were well tolerated. No adverse events were reported.

### Study Design and Data Evaluation

Table I shows the *p*-values of the potential treatment, period, sequence and subject within sequence effects obtained for the parameters AUC_(0–12)_, AUC_(0-∞) _and C_max_, which were all above 0.05. These data confirmed absence of period or sequence effects and justified pooling of the data of each of the two doses of chitosan obtained at the two different time points. The pooled data were compared to the data obtained with reference product Zovirax.

**Table I:** *P*- Values for Effects on AUC_(0-12)_, AUC_(0-∞)_ and C_max_

Source	*p*-values (*α* = 0.1)		
	AUC_(0-12)_	AUC_(0-∞)_	C_max_
Treatment	0.883	0.713	0.913
Period	0.900	0.975	0.544
Sequence	0.285	0.208	0.218
Subject within sequence	0.686	0.487	0.594

**Fig. 1:**
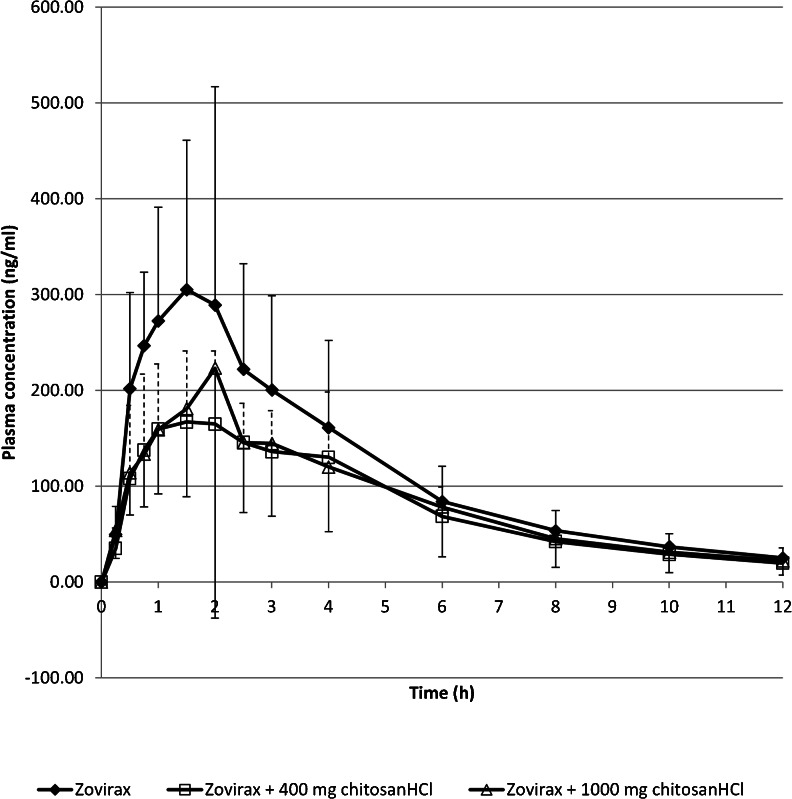
Mean acyclovir plasma profiles. For clarity of the figure, only one-way error bars representing the standard deviation are shown. *Positive bars* are shown for the Zovirax reference and for Zovirax with the 400 mg chitosan dose (*dashed line*). *Negative bars* are shown for Zovirax with the 1000 mg chitosan dose. The actual standard deviations were equal in both positive and negative direction.

**Fig. 2:**
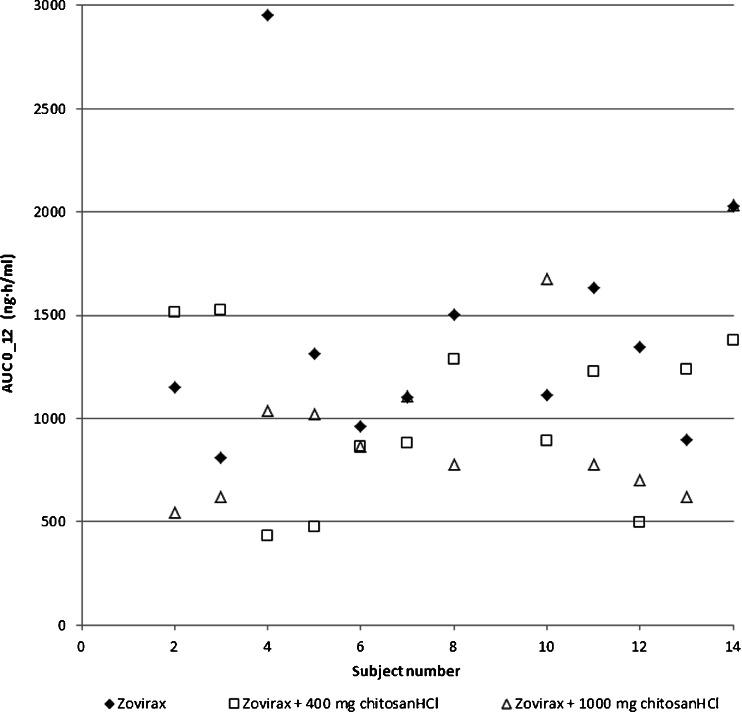
Individual data for AUC_(0-12)_.

**Fig. 3:**
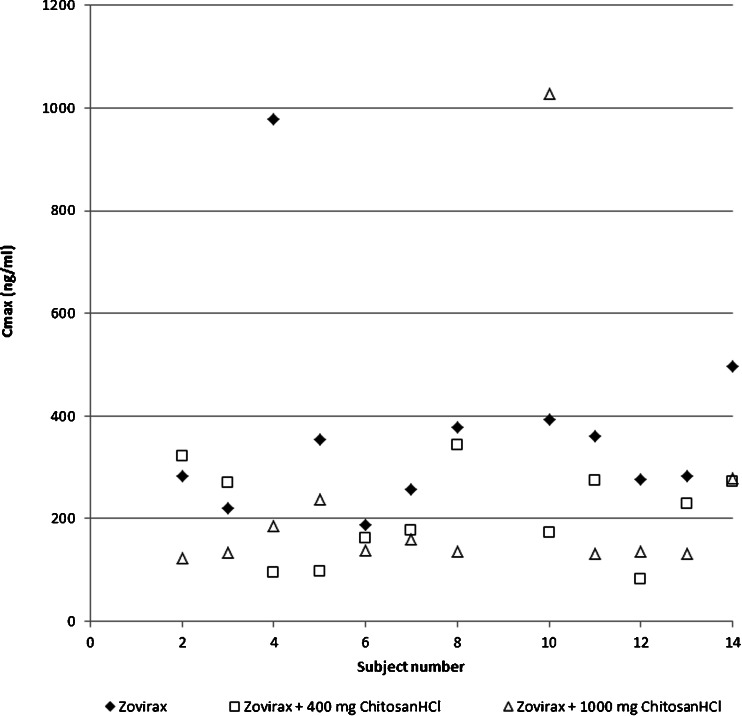
Individual data for C_max_.

Figures 1, 2, and 3 show the mean plasma curves obtained and an overview of individual data. The individual plasma profiles are accessible as supplementary material. The individual AUC data were considered sufficiently reliable as AUC_(0–12)_ was >80% AUC_(0-∞) _in all cases, except for one in a test situation with 400 mg chitosan HCl where AUC_(0–12)_ was 67% of AUC_(0-∞). _All data were used in the analysis..

### Comparative Evaluation of Pharmacokinetic Data

Table II shows the pharmacokinetic data for each of the treatments; including comparative data of previously reported pharmacokinetic data of 200 mg doses of acyclovir. C_max_, T_max_ and AUC values of the reference product were of the same magnitude as the data reported previously, both with regard to the mean values as well as to standard deviations (13,18). The mean values obtained for the C_max_ and AUC in presence of chitosan are lower than reference values reported in literature, whereas the standard deviations for C_max_ and T_max_ in presence of chitosan are higher. The elimination half-lives are also similar to those reported by others.

**Table II:** Pharmacokinetic Data (Mean Values ± sd) of a 200 mg Dose of Acyclovir, Including Comparison with Literature

Parameter	Zovirax	Zovirax + 400 mg Chitosan HCl	Zovirax + 1000 mg Chitosan HCl	Reference (1)	Reference (2)	Reference (3)
C_max_ (mg/ml)	0.373 ± 0.209	0.208 ± 0.090	0.235 ± 0.255	0.3 ± 0.1 (c, s)	0.454 ± 0.165 (t)	0.677 ± 0.209 (t)
					0.525 ± 0.205 (r)	0.707 ± 0.239 (r)
T_max_	1.2 ± 0.4	1.5 ± 0.9	1.8 ± 0.9	1.5 ± 0.6 (c)	1.6 ± 0.7 (t)	1.5 ± 0.5 (t)
(h)				1.2 ± 0.3 (s)	1.6 ± 0.7 (r)	1.5 ± 0.4 (r)
AUC_0-inf_	1.528 ± 0.627	1.132 ± 0.418	1.074 ± 0.509	1.5 ± 0.5 (c)	2.375 ± 0.739 (t)	3.422 ± 1.309 (t)
(mg · h/ml)				1.6 ± 0.5 (s)	2.632 ± 0.927 (r)	3.518 ± 1.211 (r)
				(AUC_0–24_)		
t_1/2_	3.4 ± 1.1	3.5 ± 1.0	3.2 ± 0.6	2.9 ± 0.8 (c)	3.15 ± 0.60 (t)	4.49 ± 2.37 (t)
(h)				2.9 ± 0.7 (s)	3.04 ± 0.58 (r)	3.93 ± 2.65 (r)

1. de Miranda P, Blum MR. Pharmacokinetics of acyclovir after intravenous and oral administration. J Antimicrob Chemother. 1983;12 Suppl B:29–37.

2. Vergin H, Kikuta C, Mascher H, Metz R. Pharmacokinetics and bioavailability of different formulations of aciclovir. Arzneimittelforschung. 1995;45(4):508–15.

3. Rojanasthien N, Teekachunhatean S, Kumsorn B, Chaichana N, Hay YK. Bioequivalence study of generic acyclovir compared with the brand name acyclovir. J Med Assoc Thail. 2002;85(10):1121–9.

*t* test, *r* reference tablet, *c* capsule, *s* solution

Tables III and IV list the point estimate and the 90% confidence interval for AUC and C_max_ for each of the two doses of test product *versus* the reference product. Comparison of reference acyclovir with the two situations of concomitant chitosan intake show that both the arithmetic mean of the original data and the geometric mean of the ratios (GMR) of the AUC and C_max_ decreased in presence of chitosan. The individual data show how in 9 out of 12 cases the C_max_ and AUC values of reference Zovirax are indeed higher than those of the combinations with chitosan.

**Table III:** Comparative Evaluation of 200 mg Zovirax p.o. Without (Reference) and with (Test) Concomitant 400 mg Chitosan HCl

Pharmacokinetic parameter	Geometric mean ratio test/reference	90% Confidence intervals	CV (%)
AUC_(0–12)_	0.72	0.51–1.13	46
AUC _(0-∞)_	0.73	0.54–1.00	42
Cmax	0.59	0.39–0.90	57

**Table IV:** Comparative Evaluation of 200 mg Zovirax p.o. Without (Reference) and with (Test) Concomitant 1000 mg Chitosan HCl

Pharmacokinetic parameter	Geometric mean ratio test/reference	90% Confidence intervals	CV (%)
AUC_(0–12)_	0.70	0.50–0.99	46
AUC _(0-∞)_	0.69	0.51–0.94	42
Cmax	0.58	0.38–0.88	57

The confidence intervals of the geometric mean ratios for AUC_(0–12)_ and AUC_(0-∞)_ concerning 400 mg chitosan HCl, included 1.00 and could thus not confirm a significant effect. However, those for the 1000 mg dose of chitosan did not include 1.00, pointing to a significant negative effect. The lower boundary of the 90% confidence intervals for AUC_(0–12)_ and AUC_(0-∞)_, 0.50 resp. 0.51, show how this decrease could be up to about 2-fold compared to the reference value.

For C_max_, neither 90% confidence interval includes 1.00, thereby demonstrating a significant decrease in C_max_ of acyclovir in presence of both doses of chitosan. This effect is at least 10% for the 400 mg dose and 12% for the 1000 mg dose. The confidence intervals for the 400 and 1000 mg doses almost completely overlap: the median effect is a decrease by 41–42% to values of 58–59% of the reference value (see GMR values). The decrease could be at maximum 61–62%, *i.e*. to values as low as 38–39% of the reference C_max_, considering the lower boundaries of the confidence intervals.

The T_max_ obtained with 400 mg chitosan HCl is not significantly different from the T_max_ of the reference Zovirax. However, T_max_ increased significantly in presence of 1000 mg of chitosan from 1 to 2 h (*p* = 0.029).

## DISCUSSION

The results obtained with the acyclovir reference product were in line with those published by others, both regarding the magnitude of the mean values as well as the standard deviations (see Table II).

The effects of chitosan on the bioavailability of acyclovir from Zovirax were evaluated in the context of a pretreatment with Zovirax, as administered to all volunteers on the first trial day. In view of the half-life of acyclovir of about 3 h, a carry-over effect of pretreatment with Zovirax is not expected to play a role and the order of administration of the test and reference products is not expected to affect the results (23).

Locally administered chitosan increased acyclovir’s permeability across Caco-2 membranes and facilitated the absorption of acyclovir in rats (15–17). The clinical trial protocol was based on an expected increase of C_max_ and AUC and defined that an increase by 30% would be considered as relevant. However, chitosan HCl did not act as absorption enhancer of acyclovir in the human study presented here. None of the confidence intervals includes a value of 1.3 or more, so an increase by 30% is highly unlikely.

Masuda *et al*. postulated a cut-off value of 10 kDa as minimal chain length of the chitosan polysaccharide to facilitate acyclovir’s absorption. Opanasopit *et al*. showed an increasing negative effect on the transepithelial electric resistance of Caco-2 monolayers with increasing MW in the range of 20–460 kDa (24). The applied chitosan had a MW of 30–400 kDa which is well within these ranges. Masuda *et al*. also found a dose dependent effect and the authors explained its transience by dilution of the concentration at the site of action and by neutralization of the acidified perfusate by the intestinal fluids. An enhancing effect due to changes in viscosity or cationic charges only was not found likely. Here, chitosan HCl and acyclovir were administered orally and their concentrations were diluted by the luminal fluids while progressing through the gastrointestinal tract, probably resulting in insufficiently high concentrations at acyclovir’s absorption site. Moreover, the actually dissolved and protonated fraction of chitosan may have been too low at gastrointestinal pH, thereby limiting a charge-based interaction with the epithelial membrane (25).

Chitosan is metabolized in the colon, a property that has been used to produce colon targeted formulations (26). Although evidence suggests that chitosan metabolism is likely to mainly take place in the colon, the concentration at acyclovir’s absorption site may also have been decreased by enzymatic degradation in stomach (pepsin) and/or duodenum/ileum (pancreatic enzymes) (27–32). In addition, the presence of mucus may represent an unfavorable microenvironment and have hampered the access of chitosan to the mucosa to exert its permeability enhancing effect (33,34).

If the data were to be evaluated as a bioequivalence study, a confidence interval contained within 0.8 to 1.25 would be considered as bioequivalent and confirm absence of clinically relevant difference. Considering the lower values of each of the 90% confidence intervals, which are all well below 0.8, the data do not confirm bioequivalence of the combination of Zovirax with chitosan to Zovirax only. In fact, the data unexpectedly show a reduced bioavailability of acyclovir in presence of chitosan HCl. C_max_ was significantly reduced by both chitosan doses, and AUC and T_max_ only by the 1000 mg chitosan dose. Dose dependency could not be demonstrated as point estimates for AUC and C_max_ were close and the confidence intervals for both doses of chitosan almost completely overlap. Confirmatory studies may clarify the actual significance and magnitude of the observed negative effects on the means.

Acyclovir is known for its variable absorption. De Miranda and Blum described how a 200 mg acyclovir outperformed a 200 mg acyclovir solution in as many subjects as *vice versa* thereby demonstrating how the variability in absorption is a substance related characteristic and not product related (21). Figures 2 and 3 also show variable individual effects in absence and presence of chitosan: contrary to the results of the means, three volunteers actually showed an increase of the bioavailability for one of the chitosan doses. The standard deviations calculated for AUC and C_max_ remain comparable to literature data in presence of chitosan (see Table II). However, the standard deviation for T_max_ is higher than literature values for the reference product. Several mechanisms may have influenced the bioavailability of acyclovir. Considering the effects on the variability of T_max_, the significant effects on C_max_ and AUC, and other hand, the similarity of the terminal half-lives with reference values, chitosan’s effect seems to mainly take place in the absorption phase.

Literature evidence does not point to interaction between chitosan HCl and acyclovir leading to degradation of acyclovir, either in a formulation or in the gut lumen. An effect of chitosan HCl on the dissolution (rate) of acyclovir at the time of administration is conceivable. The tablets were dispersed in 100 mL water or 100 mL chitosan solution and the glass was rinsed with another 150 mL of water. In view of acyclovir’s solubility of ≥2.3 mg/mL in pH range 1.2–7.4 at 37°C and the expected final concentration 2 mg/mL in the first 100 mL water at room temperature, not all acyclovir may have been dissolved (35,36). However, after addition of 150 ml water and assuming the presence of additional volume of aqueous fluids and a temperature of 37°C in the stomach, acyclovir was expected to dissolve sufficiently well. Experiments [data not shown] indeed confirmed the visual compatibility of 1 g chitosan HCl with 200 mg pure acyclovir after 1 min of stirring in 250 mL water at room temperature, resulting in a clear solution. In addition, any residues of undissolved acyclovir were expected to be similar for reference and test situations.

Acyclovir’s solubility is at the borderline of BCS class III and IV. Increasing its solubility in the lumen may improve its bioavailability, as demonstrated by the effects of a self-microemulsifying drug delivery system (37). *Vice versa*, its solubility may become limiting if it is chemically or physiologically reduced. Allam *et al*. showed how chitosan-acyclovir co-crystals prolonged the release of acyclovir, however, the crystal formation was not observed when acyclovir and chitosan were dissolved in 250 mL water [data not shown] (38). Nadai *et al*. showed how chitosan (25 mg/kg) prolonged T_max_ of orally administered suspensions of indomethacin and griseofulvin in rats, and reduced C_max_ and AUC. Association of the anionic indomethacin to the tertiary amino groups of chitosan did not play a role according to those authors (39). The molecular structures of chitosan (pKa ~6–6.5) and acyclovir (pKa’s 2.3 and 9.3) do not seem to favor charge based complex formation or adsorption either (40).

Effects on T_max_ and C_max_ may be related to the gastric emptying time; whereas an effect on intestinal transit time may be relevant for the absorption of the BCS class III compound acyclovir. It was previously found unlikely that chitosan caused alterations in the gastric emptying rate, as the delayed T_max_ was not observed for other compounds (39). However, in the current case, the difference in stomach content after ingestion of the chitosan dispersion (increased osmotic value, lower pH, perceived nutrient density) compared the reference dispersion may have influenced the gastric emptying rate which may explain the variable effect on C_max_ and T_max_.

Nadai *et al*. postulated an indirect effect as a cause for the prolonged T_max_: binding of bile acids to chitosan resulting in inhibition of the solubilisation of the drug substances (39). Heinen *et al*. indeed described how presence and reduction of bile salts affected the absorption of BCS class III drug trospium chloride (41). In fact, the absorption of acyclovir increased in presence of conjugated trihydroxy bile salts and bile salt-acylcarnitine mixed micelles (42,43). The 1000 mg chitosan HCl used in the clinical study presented here corresponds to about 14 mg/kg for a human being of 70 kg and is thus of the same order of magnitude as the dose used by Nadai *et al*. Although amphoteric acyclovir is in neutral state at physiological pH and is not known for having a significant food effect, chitosan may have interacted with luminal bile components indirectly resulting in a reduced bioavailability of acyclovir (14,36).

Finally, chitosan may act at the site of absorption. Chitosan’s mucoadhesive properties have been studied in potentially absorption enhancing formulations of acyclovir (44–46). Although no literature evidence is known to the authors, theoretically, chitosan may have physically or biochemically delayed or prevented access of acyclovir to its paracellular permeation route by binding to mucus at the absorption site and/or changing the microclimate *e.g*. by formation of a protective viscous layer. Derivatives of chitosan, quercetin and DM72 have been shown to inhibit the efflux of acyclovir *via* P-gp, with the aim of improving its bioavailability (47–49). An opposite effect, *i.e*. induction of (expression of) P-gp by chitosan has not been described and seems an unlikely effect of a single dose of chitosan.

An effect of chitosan on the metabolism of drugs has not been described. As acyclovir is eliminated mainly in unchanged form, such interaction is not considered likely either (14).

As chitosan is currently not in use as an excipient in approved drug products, the clinical impact of the results seems limited. However, the observed variability and complex potential interactions may complicate the use of chitosan HCl in oral pharmaceutical formulations. In addition, chitosan is advertised as food supplement in high doses, up to 6 g a day; interactions with concomitant pharmacotherapy seem possible (50).

## CONCLUSION

The results of this study in human volunteers did not confirm the data obtained in *in vitro* experiments and in rats studies that suggested that chitosan would improve the bioavailability of acyclovir. Chitosan increased the variability of the absorption of acyclovir and significantly reduced its absorption. Mechanistic studies are needed to investigate the cause for the discrepancy in these results. A luminal interaction of chitosan with acyclovir, bile salts or the epithelial membrane may be responsible for the negative effects on acyclovir’s absorption.

## Electronic supplementary material

Below is the link to the electronic supplementary material.

ESM 1 (DOCX 38 kb)

ESM 2 (PPTX 671 kb)

## Abbreviations

(HP)LC: (High pressure) liquid chromatography
AUC: Area under the curve
AUC: Area under the curve
BCS: Biopharmaceutics classification system
CI: Confidence interval
Cmax: Maximum plasma concentration
CV: Coefficient of variation
DD: Degree of deacetylation
EMA: European Medicines Agency
GMR: Geometric mean ratio
HCl: Hydrochloride
kDa: kiloDalton
MS: Mass spectrometry
MW: Molecular weight
P-gp: P-glycoprotein
Ph.Eur.: European Pharmacopoeia
Tmax: Time point at which the maximum concentration is measured
