# Supplementary material for: The Influence of Chitosan on the Oral Bioavailability of Acyclovir—a Comparative Bioavailability Study in Humans
Source: Pharm Res. 2015 Jan 22;32(7):2241–9. doi: 10.1007/s11095-014-1613-y (PMC4452255; doi:10.1007/s11095-014-1613-y)
Supplement: Supplementary file 1 — (DOCX 38 kb) [file 11095_2014_1613_MOESM1_ESM.docx]

**Supplementary material 1 - Bioanalytical method validation**

**Description**

The bioanalytical method was modified from previously reported methods ([1-3](#_ENREF_1)). Plasma samples of 500µl were prepared for analysis by solid phase extraction, using Oasis HLB 1cc cartridges (30mg) supplied by Waters. The column was washed with 500ml water and then eluted with 500µl acetonitrile. The extract was centrifuged at 14000 rpm for 10 minutes at -5°C and the concentration of acyclovir was determined by LC-MS/MS using a sample injection volume of 10 µl. The HPLC consisted of a Prontosil C18; 100*2,00mm; 5µm column, using an Agilent 1100 LC binary pump. A gradient elution was used with mobile phase A and B where A consisted of 15mM ammonium acetate + 0.1375% formic acid at pH 3.5 and B was acetonitrile + 0.1375% formic acid. Details of the gradient:

| Step | Total Time(min) | Flow Rate(µl/min) | A (%) | B (%) |
| --- | --- | --- | --- | --- |
| 0 | 0.00 | 300 | 97.0 | 3.0 |
| 1 | 0.50 | 300 | 97.0 | 3.0 |
| 2 | 0.60 | 300 | 5.0 | 95.0 |
| 3 | 1.20 | 300 | 5.0 | 95.0 |
| 4 | 2.00 | 300 | 97.0 | 3.0 |
| 5 | 5.50 | 300 | 97.0 | 3.0 |

Detection took place by a triple quadrupole LC-MS/MS mass spectrometer, API 3000 manufactured by AB Sciex Instruments, using multiple reactions monitoring with transitions Q1/Q3: 255,992→ 151,873. Source temperature was 500°C, overall run time was 5.5 minutes. The method was linear in a range of 10 to 800 ng/ml, with a detection limit of 1 ng/ml. QC samples were analyzed with the plasma samples to monitor method accuracy and precision.

**Table 1 Summary of bioanalytical method validation data**

| Short description of the method | HPLC/MS/MS – see above |
| --- | --- |
| Biological matrix | Plasma |
| Analyte | Acyclovir |
| Internal standard (IS) | See selectivity |
| Calibration concentrations (ng/ml) – 10; 12.5; 25; 50; 200; 400; 800 | |
| Lower limit of quantification (ng/ml) | 10 ng/ml, accuracy 106%, CV 26.0% |
| QC concentrations (ng/ml) | |
| Between-run accuracy | By QC |
| Between-run precision | By QC |
| Within-run accuracy | By QC |
| Within-run precision | By QC |
| Matrix Factor (MF) (all QC) | Not applicable |
| Short term stability of the stock solution and working solutions | By QC |
| Short term stability in biological matrix at room temperature or at sample processing temperature. | By QC |
| Long term stability in biological matrix | See short term stability |
| Autosampler storage stability | By QC |
| Post-preparative stability | By QC |
| Freeze and thaw stability | Confirmed by literature ([1-3](#_ENREF_1)) |

**Selectivity**

The analytical method is able to differentiate acyclovir from endogenous components in the matrix or other components in the sample. Ganciclovir was added to the samples as optional internal standard. However, the CV and correlation coefficient of the method were not improved by addition of ganciclovir. Calculation of acyclovir concentrations was therefore performed not taking into account the ganciclovir. No other interfering peaks were observed.

**Carry-over**

Calibration curves were obtained using increasing and decreasing concentration ranges. Accuracy and precision remained acceptable independent of the order of injection. No carry-over was observed.

**Calibration curve**

The back calculated concentrations of the calibration standards/QC samples were generally within 15% of the nominal value. The intraday precision data in Table 2 showed a CV<15% for 6 out of 7 concentrations, with a CV 26% for the LLOQ, while the interday precision (11 days) data in Table 3 showed a CV <15% for 6 out of 7 concentrations and CV<20% for one concentration. This was considered acceptable.

**Table 2 Intraday precision and accuracy***

| Nominal Concentration [ng/ml] | Number of Values | MeanCalculated Concentration | Accuracy [%] | Std. Deviation | %CV |
| --- | --- | --- | --- | --- | --- |
| 10 | 3 | 10.64 | 106.4 | 2.77 | 26.0 |
| 12.5 | 3 | 10.48 | 83.8 | 1.54 | 14.7 |
| 25 | 3 | 25.24 | 101.0 | 3.34 | 13.2 |
| 50 | 3 | 47.53 | 95.1 | 3.58 | 7.5 |
| 200 | 3 | 191.33 | 95.7 | 30.79 | 16.1 |
| 400 | 3 | 413.49 | 103.4 | 25.96 | 6.3 |
| 800 | 3 | 795.59 | 99.4 | 59.82 | 7.5 |

*overall regression equation: y = 0.0123 x (r = 0.9978); ascending 1: y = 0.0114 x (r = 0.9980); descending: y = 0.0131 x (r = 0.9998); ascending 2: y = 0.0124 x (r = 0.9997)

**Table 3 Interday precision and accuracy**

| Nominal Concentration [ng/ml] | Mean calculated concentrationat day 1-11 | Accuracy [%] | Std. Deviation | %CV |
| --- | --- | --- | --- | --- |
| 10.0 | 10.12 | 101.15 | 0.39 | 3.85 |
| 12.5 | 9.99 | 79.90 | 0.90 | 8.98 |
| 25.0 | 19.93 | 79.73 | 3.68 | 18.46 |
| 50.0 | 49.38 | 98.76 | 1.80 | 3.65 |
| 200.0 | 204.53 | 102.27 | 26.27 | 12.85 |
| 400.0 | 406.23 | 101.56 | 1.93 | 0.47 |
| 800.0 | 797.40 | 99.68 | 5.83 | 0.73 |

**Accuracy**

Within-run/intraday accuracy

See Table 2. Within-run accuracy was determined by analyzing in a single run 3 samples per level at 7 concentration levels covering the calibration curve range. The mean concentration was well within 15% of the nominal values for the QC samples, except for the LLOQ which was within 26% of the nominal value. Considering the other lower values this was considered sufficient for this study.

Between-run/interday accuracy

See Table 3. For the validation of the between-run accuracy, LLOQ, low, medium and high QC samples from at least seven runs analysed on a total of 11 different days were evaluated. The mean concentration was generally well within 15% of the nominal values for the QC samples, except for 12.5 and 25.0 ng/ml which remained within 20% of the nominal value.

**Precision**

Within-run precision

For the validation of the within-run precision, there should be a minimum of five samples per concentration level at LLOQ, low, medium and high QC samples in a single run. The within-run CV value should not exceed 15% for the QC samples, except for the LLOQ which should not exceed 20%.

Between –run precision

For the validation of the between-run precision, LLOQ, low, medium and high QC samples from at least three runs analysed on at least two different days should be evaluated. The between-run CV value should not exceed 15% for the QC samples, except for the LLOQ which should not exceed 20%.

**Matrix effect**

Matrix effects of plasma were excluded by comparison of samples of two different donors which both resulted in calibration curves with individual and mean r > 0.999 and acceptable CV and accuracy values; see also Table 4.

**Table 4 Matrix effects in two different donor plasma samples**

| Expected Concentration [ng/ml] | Number of Values | MeanCalculated Concentration | % Accuracy | Std. Deviation | %CV | |
| --- | --- | --- | --- | --- | --- | --- |
| 6.25 | 2 | 7.21 | 115.4 | 0.04 | | 0.6 |
| 12.5 | 2 | 13.02 | 104.2 | 1.65 | | 12.7 |
| 25 | 2 | 25.68 | 102.7 | 0.04 | | 0.2 |
| 50 | 2 | 50.39 | 100.8 | 8.54 | | 17.0 |
| 100 | 2 | 102.97 | 103.0 | 1.50 | | 1.5 |
| 200 | 2 | 198.27 | 99.1 | 3.74 | | 1.9 |

**Literature references**

1. Tian Y, Lin H, Zhang XY, Zhang ZJ, Mao GG. Rapid quantification of the metabolite of valacyclovir hydrochloride in human plasma by liquid chromatography-tandem mass spectrometry. Academic Journal of Xi'an Jiaotong University. 2010;22(2):83-90.

2. Kanneti R, Rajesh R, Aravinda Raj JR, Bhatt PA. An LC-MS-MS method for the simultaneous quantitation of acyclovir and valacyclovir in human plasma. Chromatographia. 2009;70(3-4):407-14.

3. Merzlikine A, Rotter C, Rago B, Poe J, Christoffersen C, Thomas VH, et al. Effect of chitosan glutamate, carbomer 974P, and EDTA on the in vitro Caco-2 permeability and oral pharmacokinetic profile of acyclovir in rats. Drug Dev Ind Pharm. 2009;35(9):1082-91.
