# Supplementary material for: The Influence of Chitosan on the Oral Bioavailability of Acyclovir—a Comparative Bioavailability Study in Humans
Source: Pharm Res. 2015 Jan 22;32(7):2241–9. doi: 10.1007/s11095-014-1613-y (PMC4452255; doi:10.1007/s11095-014-1613-y)
Supplement: Supplementary file 2 — (PPTX 671 kb) [file 11095_2014_1613_MOESM2_ESM.pptx]

## Slide 1
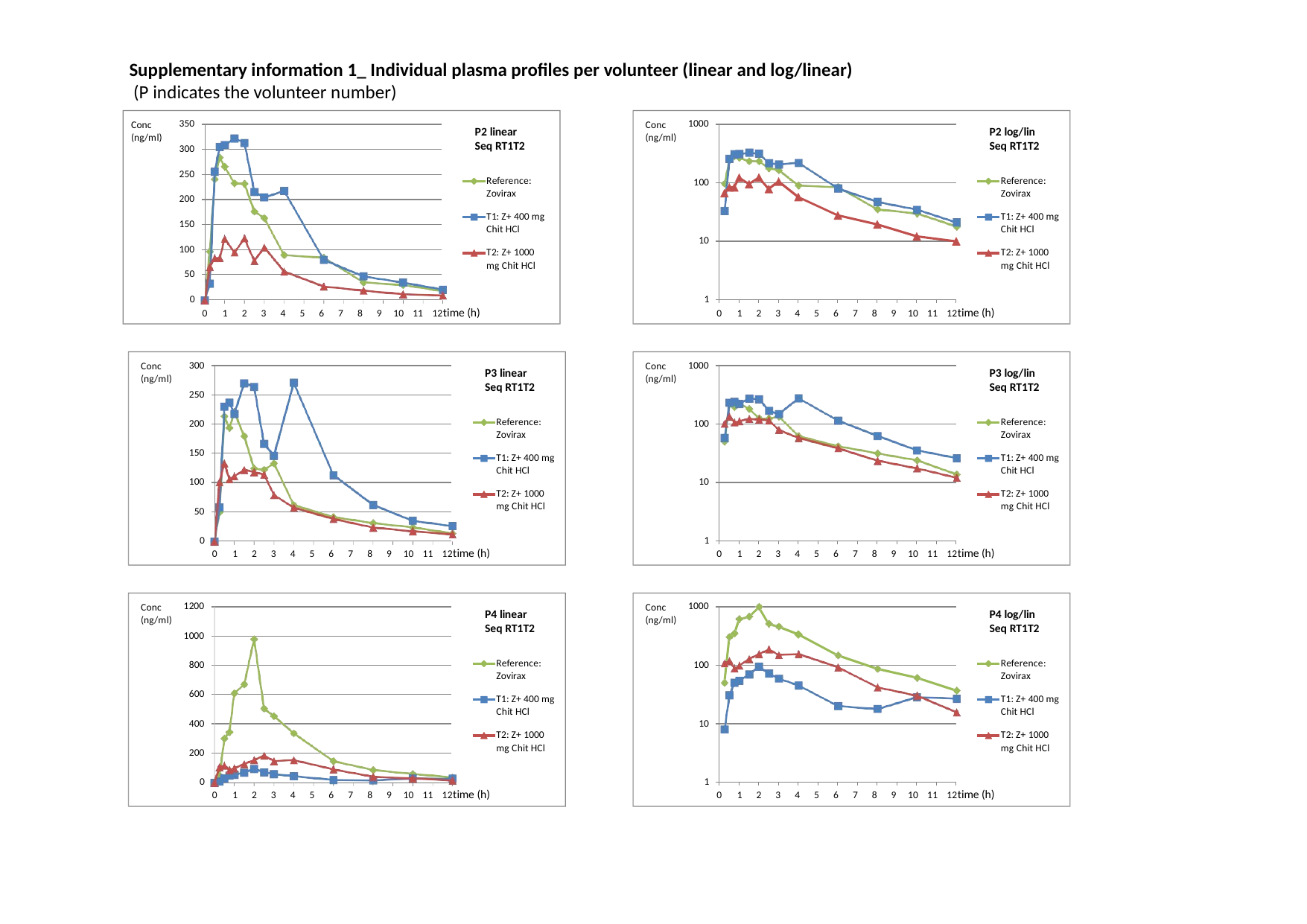

Supplementary information 1_ Individual plasma profiles per volunteer (linear and log/linear)
 (P indicates the volunteer number)
1000
350
Conc (ng/ml)
Conc (ng/ml)
P2 linear Seq RT1T2
P2 log/lin Seq RT1T2
300
250
Reference: Zovirax
Reference: Zovirax
100
200
T1: Z+ 400 mg
Chit HCl
T1: Z+ 400 mg
Chit HCl
150
10
100
T2: Z+ 1000
mg Chit HCl
T2: Z+ 1000
mg Chit HCl
50
0
1
0 1 2 3 4 5 6 7 8 9 10 11 12time (h)
0 1 2 3 4 5 6 7 8 9 10 11 12time (h)
300
1000
Conc (ng/ml)
Conc (ng/ml)
P3 linear Seq RT1T2
P3 log/lin Seq RT1T2
250
Reference: Zovirax
Reference: Zovirax
200
100
150
T1: Z+ 400 mg
Chit HCl
T1: Z+ 400 mg
Chit HCl
100
10
T2: Z+ 1000
mg Chit HCl
T2: Z+ 1000
mg Chit HCl
50
0
1
0 1 2 3 4 5 6 7 8 9 10 11 12time (h)
0 1 2 3 4 5 6 7 8 9 10 11 12time (h)
1200
1000
Conc (ng/ml)
Conc (ng/ml)
P4 linear Seq RT1T2
P4 log/lin Seq RT1T2
1000
Reference: Zovirax
Reference: Zovirax
800
100
600
T1: Z+ 400 mg
Chit HCl
T1: Z+ 400 mg
Chit HCl
400
10
T2: Z+ 1000
mg Chit HCl
T2: Z+ 1000
mg Chit HCl
200
0
1
0 1 2 3 4 5 6 7 8 9 10 11 12time (h)
0 1 2 3 4 5 6 7 8 9 10 11 12time (h)

## Slide 2
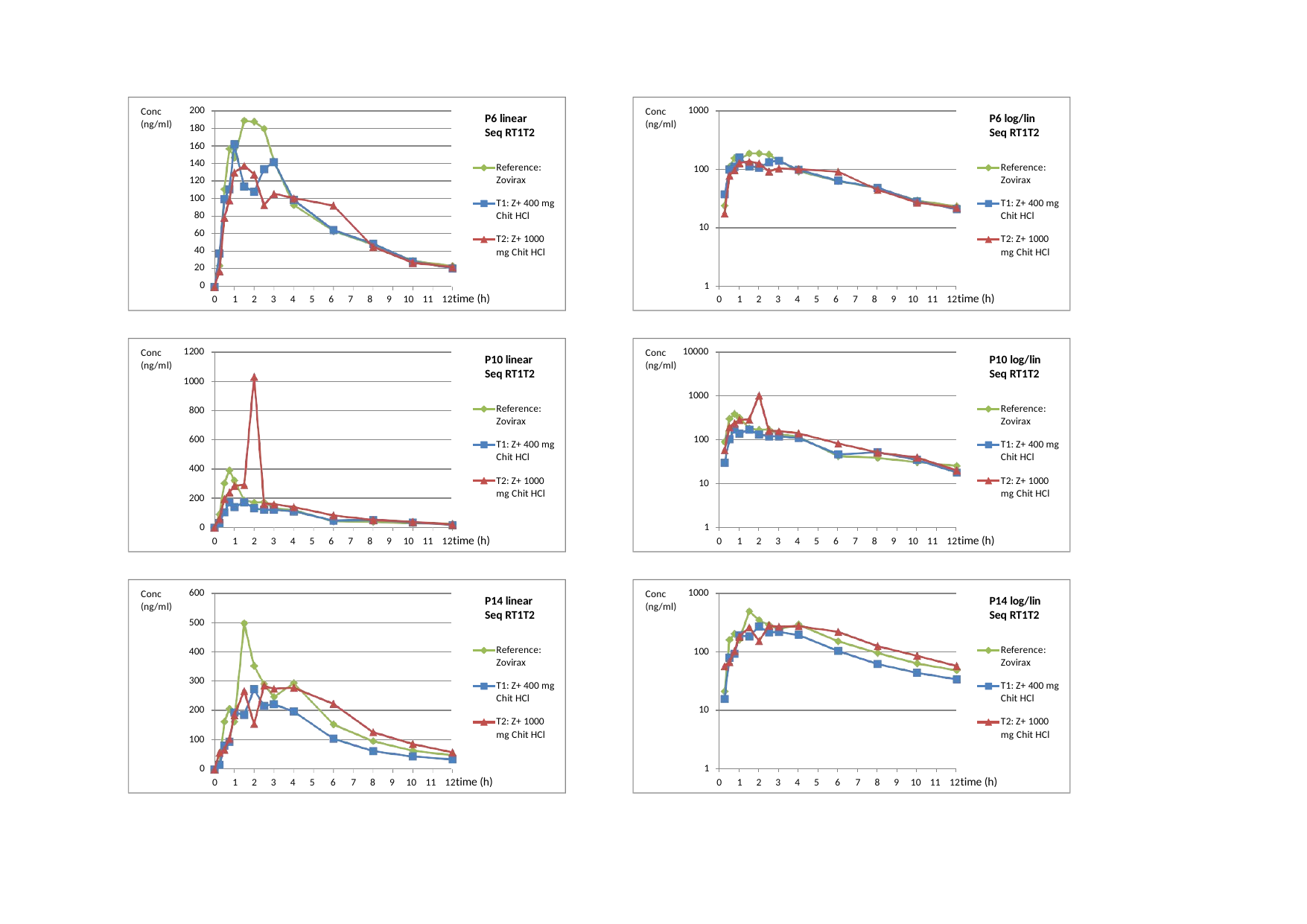

1000
200
180
160
140
120
100
80
60
40
20
0
Conc (ng/ml)
Conc (ng/ml)
P6 linear Seq RT1T2
P6 log/lin Seq RT1T2
Reference: Zovirax
Reference: Zovirax
100
T1: Z+ 400 mg
Chit HCl
T1: Z+ 400 mg
Chit HCl
10
T2: Z+ 1000
mg Chit HCl
T2: Z+ 1000
mg Chit HCl
1
0 1 2 3 4 5 6 7 8 9 10 11 12time (h)
0 1 2 3 4 5 6 7 8 9 10 11 12time (h)
1200
10000
Conc (ng/ml)
Conc (ng/ml)
P10 linear Seq RT1T2
P10 log/lin Seq RT1T2
1000
1000
Reference: Zovirax
Reference: Zovirax
800
600
100
T1: Z+ 400 mg
Chit HCl
T1: Z+ 400 mg
Chit HCl
400
T2: Z+ 1000
mg Chit HCl
T2: Z+ 1000
mg Chit HCl
10
200
0
1
0 1 2 3 4 5 6 7 8 9 10 11 12time (h)
0 1 2 3 4 5 6 7 8 9 10 11 12time (h)
600
1000
Conc (ng/ml)
Conc (ng/ml)
P14 linear Seq RT1T2
P14 log/lin Seq RT1T2
500
Reference: Zovirax
Reference: Zovirax
400
100
300
T1: Z+ 400 mg
Chit HCl
T1: Z+ 400 mg
Chit HCl
200
10
T2: Z+ 1000
mg Chit HCl
T2: Z+ 1000
mg Chit HCl
100
0
1
6 7 8 9 10 11 12time (h)
6 7 8 9 10 11 12time (h)
0 1 2 3 4 5
0 1 2 3 4 5

## Slide 3
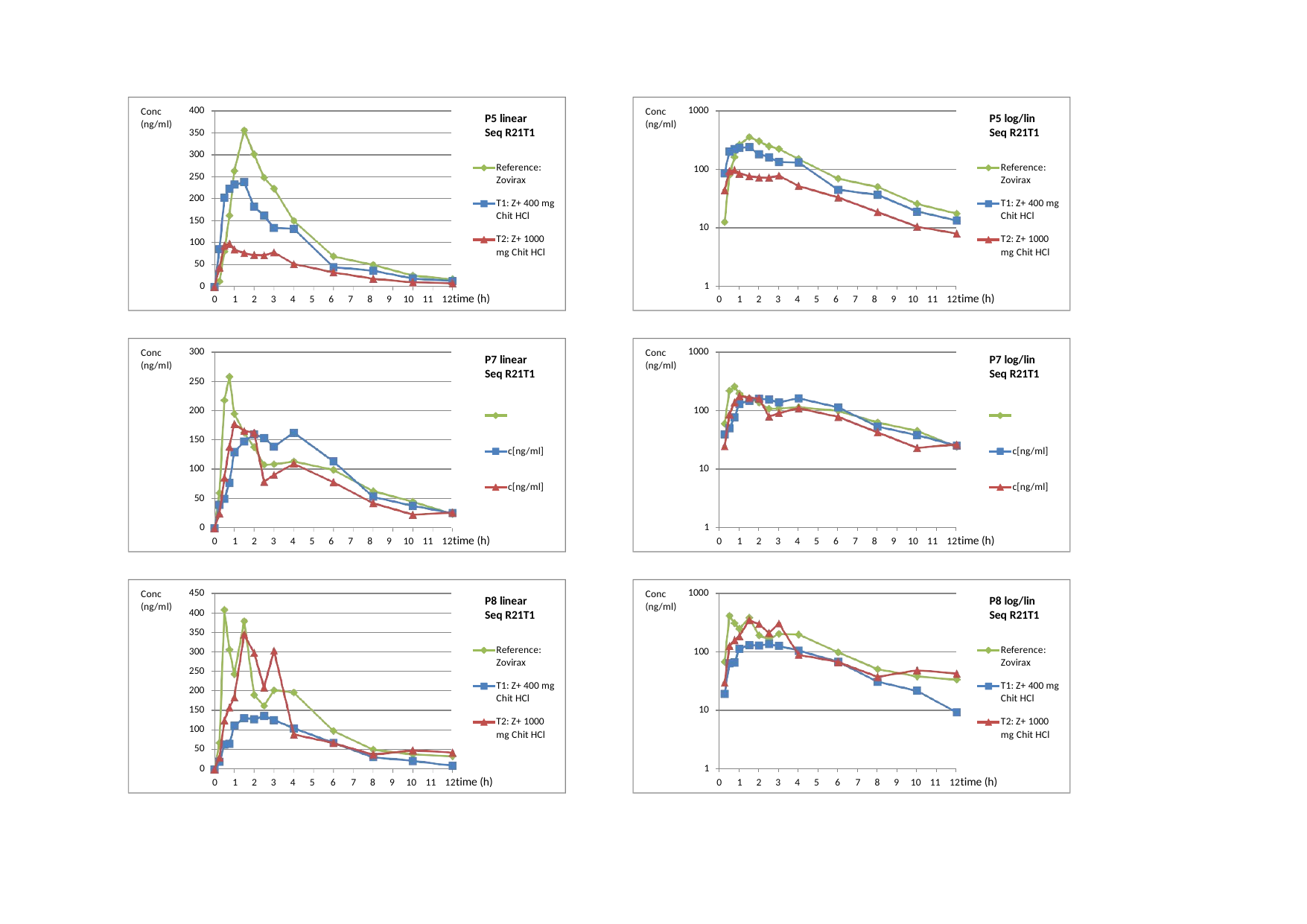

400
350
300
1000
Conc (ng/ml)
Conc (ng/ml)
P5 linear Seq R21T1
P5 log/lin Seq R21T1
Reference: Zovirax
Reference: Zovirax
100
250
200
T1: Z+ 400 mg
Chit HCl
T1: Z+ 400 mg
Chit HCl
150
10
T2: Z+ 1000
mg Chit HCl
T2: Z+ 1000
mg Chit HCl
100
50
0
1
0 1 2 3 4 5 6 7 8 9 10 11 12time (h)
0 1 2 3 4 5 6 7 8 9 10 11 12time (h)
300
1000
Conc (ng/ml)
Conc (ng/ml)
P7 linear Seq R21T1
P7 log/lin Seq R21T1
250
200
100
150
c[ng/ml]
c[ng/ml]
100
10
c[ng/ml]
c[ng/ml]
50
0
1
0 1 2 3 4 5 6 7 8 9 10 11 12time (h)
0 1 2 3 4 5 6 7 8 9 10 11 12time (h)
450
400
350
300
250
200
150
100
50
1000
Conc (ng/ml)
Conc (ng/ml)
P8 linear Seq R21T1
P8 log/lin Seq R21T1
Reference: Zovirax
Reference: Zovirax
100
T1: Z+ 400 mg
Chit HCl
T1: Z+ 400 mg
Chit HCl
10
T2: Z+ 1000
mg Chit HCl
T2: Z+ 1000
mg Chit HCl
0
1
6 7 8 9 10 11 12time (h)
6 7 8 9 10 11 12time (h)
0 1 2 3 4 5
0 1 2 3 4 5

## Slide 4
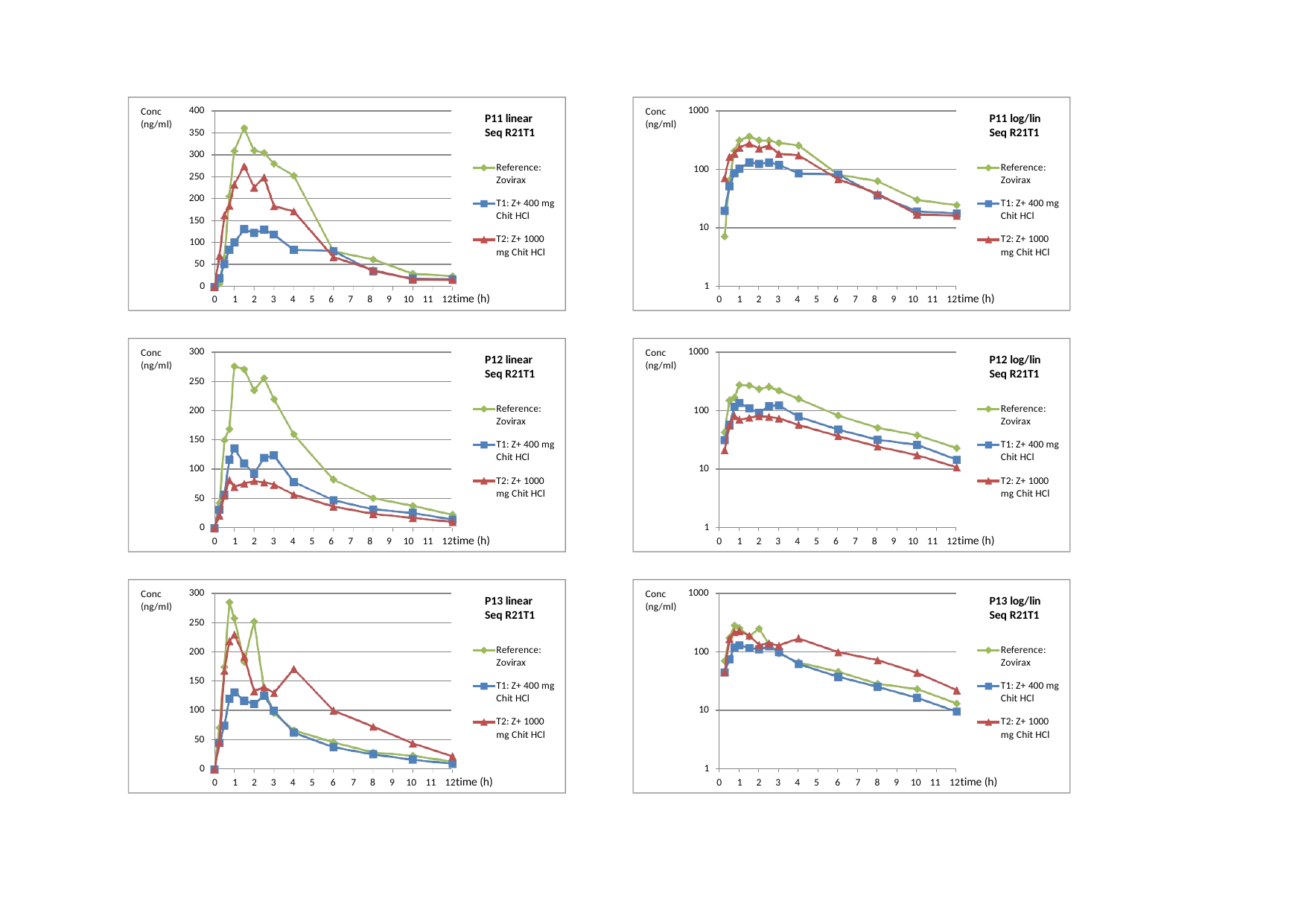

400
350
300
1000
Conc (ng/ml)
Conc (ng/ml)
P11 linear Seq R21T1
P11 log/lin Seq R21T1
Reference: Zovirax
Reference: Zovirax
100
250
200
T1: Z+ 400 mg
Chit HCl
T1: Z+ 400 mg
Chit HCl
150
10
T2: Z+ 1000
mg Chit HCl
T2: Z+ 1000
mg Chit HCl
100
50
0
1
0 1 2 3 4 5 6 7 8 9 10 11 12time (h)
0 1 2 3 4 5 6 7 8 9 10 11 12time (h)
300
1000
Conc (ng/ml)
Conc (ng/ml)
P12 linear Seq R21T1
P12 log/lin Seq R21T1
250
Reference: Zovirax
Reference: Zovirax
200
100
150
T1: Z+ 400 mg
Chit HCl
T1: Z+ 400 mg
Chit HCl
100
10
T2: Z+ 1000
mg Chit HCl
T2: Z+ 1000
mg Chit HCl
50
0
1
0 1 2 3 4 5 6 7 8 9 10 11 12time (h)
0 1 2 3 4 5 6 7 8 9 10 11 12time (h)
300
1000
Conc (ng/ml)
Conc (ng/ml)
P13 linear Seq R21T1
P13 log/lin Seq R21T1
250
Reference: Zovirax
Reference: Zovirax
200
100
150
T1: Z+ 400 mg
Chit HCl
T1: Z+ 400 mg
Chit HCl
100
10
T2: Z+ 1000
mg Chit HCl
T2: Z+ 1000
mg Chit HCl
50
0
1
6 7 8 9 10 11 12time (h)
6 7 8 9 10 11 12time (h)
0 1 2 3 4 5
0 1 2 3 4 5
